# Caesium europium(III) polyphosphate, CsEu(PO_3_)_4_

**DOI:** 10.1107/S1600536809036058

**Published:** 2009-09-12

**Authors:** Jing Zhu, Wen-Dan Cheng, Hao Zhang

**Affiliations:** a Department of Materials Science and Engineering, Yunnan University, Kunming, Yunnan 650091, People’s Republic of China; b State Key Laboratory of Structural Chemistry, Fujian Institute of Research on the Structure of Matter, Chinese Academy of Sciences, Fuzhou, Fujian 350002, People’s Republic of China

## Abstract

Caesium europium polyphosphate, CsEu(PO_3_)_4_, was synthesized by a high-temperature solution reaction. Its structure is charaterized by a three-dimensional framework made up of double PO_4_ spiral chains and EuO_8_ and CsO_11_ polyhedra.

## Related literature

For the structures, properties and applications of condensed alkaline metal–rare earth polyphosphates with the general formula *MLn*(PO_3_)_4_ (*M* = alkali metal, *Ln* = rare earth metal), see: Chinn & Hong (1975 ▶); Ettis *et al.* (2003 ▶); Hong (1975 ▶); Koizumi (1976 ▶); Koizumi & Nakano (1978 ▶); Maksimova *et al.* (1982 ▶); Naïli & Mhiri (2005 ▶); Otsuka *et al.* (1977 ▶); Palkina *et al.* (1978 ▶); Rekik *et al.* (2004 ▶); Tsujimoto *et al.* (1977 ▶).

## Experimental

### 

#### Crystal data

CsEu(PO_3_)_4_
*M* *_r_* = 600.75
Monoclinic, 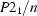

*a* = 10.3571 (9) Å
*b* = 8.9615 (5) Å
*c* = 11.1957 (8) Å
β = 106.354 (3)°
*V* = 997.09 (13) Å^3^
*Z* = 4
Mo *K*α radiation
μ = 10.60 mm^−1^
*T* = 293 K
0.25 × 0.20 × 0.15 mm

#### Data collection

Bruker P4 diffractometer
Absorption correction: ψ-scan (*XSCANS*; Bruker, 1996 ▶) *T*_min_ = 0.545, *T*_max_ = 1.000
7446 measured reflections
2284 independent reflections
2185 reflections with *I* > 2σ(*I*)
*R*_int_ = 0.025
3 standard reflections every 97 reflections intensity decay: none

#### Refinement

*R*[*F* ^2^ > 2σ(*F* ^2^)] = 0.019
*wR*(*F* ^2^) = 0.048
*S* = 1.00
2284 reflections
164 parameters
Δρ_max_ = 1.16 e Å^−3^
Δρ_min_ = −1.17 e Å^−3^

### 

Data collection: *XSCANS* (Bruker, 1996 ▶); cell refinement: *XSCANS*; data reduction: *SHELXTL* (Sheldrick, 2008 ▶); program(s) used to solve structure: *SHELXS97* (Sheldrick, 2008 ▶); program(s) used to refine structure: *SHELXL97* (Sheldrick, 2008 ▶); molecular graphics: *SHELXTL*; software used to prepare material for publication: *SHELXTL*.

## Supplementary Material

Crystal structure: contains datablocks I. DOI: 10.1107/S1600536809036058/bt5050sup1.cif

Structure factors: contains datablocks I. DOI: 10.1107/S1600536809036058/bt5050Isup2.hkl

Additional supplementary materials:  crystallographic information; 3D view; checkCIF report

## Figures and Tables

**Fig. 1.**
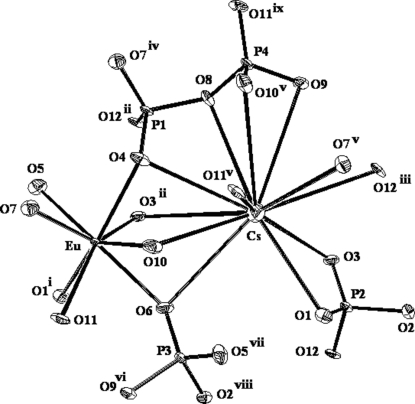
Displacement ellipsoid plot (50% probability) of CsEu(PO3)4.

## supplementary crystallographic information

### Comment

Condensed alkaline metal-rare earth polyphosphates with the general formula MLn(PO_3_)_4_ (*M* = alkali metal, Ln = rare earth metal) possess various structures (Ettis *et al.*, 2003; Rekik *et al.*, 2004) and desirable optical properties (Chinn *et al.*, 1975; Otsuka *et al.*, 1977; Tsujimoto *et al.*, 1977; Hong *et al.*, 1975; Koizumi *et al.*, 1976). Furthermore, their chemical and thermal stability ensures the feasibility of the industrial applications. For this reason some polyphosphates (Naïli *et al.*, 2005; Palkina *et al.*, 1978; Maksimova *et al.*, 1982) were synthesized and investigated in the Cs–Ln–P–O system as an important potential. However, polyphosphates containing europium have not to date been fully explored in the system. Our exploration on the system afforded caesium europium polyphosphate CsEu(PO_3_)_4_. We report herein the synthesis and crystal structure of CsEu(PO_3_)_4_.

Crystallographic data and structural refinement of CsEu(PO_3_)_4_ are summarized in Table 1. The atomic coordinates and thermal parameters are listed in Table 2. Selected bond lengths and angles are given in Table 3.

The structure of crystal CsEu(PO_3_)_4_ is shown in Fig. 1. It belongs to the monoclinic space group *P*2_1_/*n*, which is isostructural with CsGd(PO_3_)_4_ (Naïli *et al.*, 2005). The crystallographically distinct atoms of the asymmetric unit in the structure are one caesium, one europium, four phosphorus, and twelve oxygen atoms. It is described as a three-dimensional framework made up from double PO_4_ spiral chains and Cs- and Eu-polyhedra. As illustrated in Fig. 2, the double PO_4_ spiral chains have the same repeating unit (eight PO_4_ tetrahedra) as single one of CsNd(PO_3_)_4_ (Koizumi *et al.*, 1978). These spiral chains are linked by EuO_8_ and CsO_11_ polyhedra. In addition, comparing with CsNd(PO_3_)_4_, the different characteristics are noted in the coordination environments of the cations. The Eu cation is eight-coordinated with the Eu—O band distances ranging from 2.344 (3) to 2.471 (3) Å. Each EuO_8_ polyhedron is corner- and face-connected with two and two CsO_11_ polyhedra, respectively (Fig. 3). The isolation of EuO_8_ polyhedra gives rise to the large Eu—Eu distances, the shortest of which is 5.7415 (3) Å. The Cs cation is coordinated by eleven oxygen atoms, and the large range of the Cs—O bond distances implies that CsO_11_ polyhedra are distorted. Neighboring two CsO_11_ polyhedra are linked by corner-sharing (Fig. 4).

### Experimental

The title compound was prepared by the high temperature solution reaction, using analytical reagents Cs_2_CO_3_, Eu_2_O_3_, and NH_4_H_2_PO_4_ in the molar ratio of Cs/Eu/P = 7:1:12. Starting mixtures were finely ground in an agate mortar to ensure the best homogeneity and reactivity, then placed in a platinum crucible and heated at 373 K for 4 h. Afterwards, the mixtures were reground and heated to 973 K for 24 h. Finally, the temperature was cooled to 773 K at a rate of 2 K/h and air-quenched to room temperature. A few colorless and block-shaped crystals were obtained from the melt of the mixture.

### Refinement

A single-crystal of the compound was selected for X-ray Diffraction determination. The structure was solved using direct methods and refined on F2 by the full-matrix least-squares method with the *SHELXL97* program package (Sheldrick, 2008). The position of the Eu atom was refined by the application of the direct method, and the remaining atoms were located in succeeding difference Fourier synthesis. In order to confirm the chemical composition of the the compound, the single-crystal investigated on the diffractometer was analyzed by Energy-dispersive X-ray spectrometry (EDX) using a JSM6700F scanning electron microscope. The obtained result is in good agreement with that obtained by the refinement of the crystal structure. No impurity elements have been detected.

### Crystal data

**Table:**

CsEu(PO_3_)_4_	*F*(000) = 1096
*M**_r_* = 600.75	*D*_x_ = 4.002 Mg m^−^^3^
Monoclinic, *P*2_1_/*n*	Mo *K*α radiation, λ = 0.71073 Å
Hall symbol: -P 2yn	Cell parameters from 2827 reflections
*a* = 10.3571 (9) Å	θ = 2.3–27.5°
*b* = 8.9615 (5) Å	µ = 10.60 mm^−^^1^
*c* = 11.1957 (8) Å	*T* = 293 K
β = 106.354 (3)°	Prism, colorless
*V* = 997.09 (13) Å^3^	0.25 × 0.20 × 0.15 mm
*Z* = 4	

### Data collection

**Table:**

Bruker P4 diffractometer	2284 independent reflections
Radiation source: fine-focus sealed tube	2185 reflections with *I* > 2σ(*I*)
graphite	*R*_int_ = 0.025
Detector resolution: 14.6306 pixels mm^-1^	θ_max_ = 27.5°, θ_min_ = 2.4°
ω scans	*h* = −10→13
Absorption correction: ψ scan (*XSCANS*; Bruker, 1996)	*k* = −10→11
*T*_min_ = 0.545, *T*_max_ = 1.000	*l* = −14→14
7446 measured reflections	

### Refinement

**Table:**

Refinement on *F*^2^	Primary atom site location: structure-invariant direct methods
Least-squares matrix: full	Secondary atom site location: difference Fourier map
*R*[*F*^2^ > 2σ(*F*^2^)] = 0.019	*w* = 1/[σ^2^(*F*_o_^2^) + (0.0273*P*)^2^ + 2.176*P*] where *P* = (*F*_o_^2^ + 2*F*_c_^2^)/3
*wR*(*F*^2^) = 0.048	(Δ/σ)_max_ = 0.001
*S* = 1.00	Δρ_max_ = 1.16 e Å^−^^3^
2284 reflections	Δρ_min_ = −1.17 e Å^−^^3^
164 parameters	Extinction correction: *SHELXL97* (Sheldrick, 2008), Fc^*^=kFc[1+0.001xFc^2^λ^3^/sin(2θ)]^-1/4^
0 restraints	Extinction coefficient: 0.0154 (3)

### Special details

**Table:**

Geometry. All e.s.d.'s (except the e.s.d. in the dihedral angle between two l.s. planes) are estimated using the full covariance matrix. The cell e.s.d.'s are taken into account individually in the estimation of e.s.d.'s in distances, angles and torsion angles; correlations between e.s.d.'s in cell parameters are only used when they are defined by crystal symmetry. An approximate (isotropic) treatment of cell e.s.d.'s is used for estimating e.s.d.'s involving l.s. planes.
Refinement. Refinement of *F*^2^ against ALL reflections. The weighted *R*-factor *wR* and goodness of fit *S* are based on *F*^2^, conventional *R*-factors *R* are based on *F*, with *F* set to zero for negative *F*^2^. The threshold expression of *F*^2^ > σ(*F*^2^) is used only for calculating *R*-factors(gt) *etc*. and is not relevant to the choice of reflections for refinement. *R*-factors based on *F*^2^ are statistically about twice as large as those based on *F*, and *R*- factors based on ALL data will be even larger.

### Fractional atomic coordinates and isotropic or equivalent isotropic displacement parameters (Å^2^)

**Table:**

	*x*	*y*	*z*	*U*_iso_*/*U*_eq_	
Eu	0.498050 (16)	0.226449 (17)	0.180852 (15)	0.00580 (8)	
Cs	0.67980 (2)	−0.06549 (3)	0.45979 (2)	0.01775 (9)	
P1	0.45959 (9)	−0.17482 (9)	0.13333 (8)	0.00609 (17)	
P2	0.75501 (8)	0.02838 (9)	0.78552 (8)	0.00606 (17)	
P3	0.67259 (9)	0.39350 (9)	0.47427 (8)	0.00652 (17)	
P4	0.64481 (9)	−0.40738 (9)	0.25829 (8)	0.00669 (17)	
O1	0.8311 (3)	0.0968 (3)	0.7052 (2)	0.0103 (5)	
O2	0.8623 (2)	−0.0428 (3)	0.9043 (2)	0.0084 (5)	
O3	0.6485 (3)	−0.0823 (3)	0.7252 (2)	0.0097 (5)	
O4	0.5366 (2)	−0.0336 (3)	0.1654 (2)	0.0112 (5)	
O5	0.3125 (3)	0.1627 (3)	0.0137 (2)	0.0122 (5)	
O6	0.5645 (3)	0.2902 (3)	0.4047 (2)	0.0110 (5)	
O7	0.5603 (3)	0.2458 (3)	−0.0102 (2)	0.0110 (5)	
O8	0.5213 (2)	−0.2937 (3)	0.2424 (2)	0.0097 (5)	
O9	0.6665 (3)	−0.4514 (3)	0.4006 (2)	0.0116 (5)	
O10	0.7367 (2)	0.1781 (3)	0.2529 (2)	0.0120 (5)	
O11	0.6003 (3)	0.4610 (3)	0.1767 (2)	0.0115 (5)	
O12	0.6881 (2)	0.1588 (3)	0.8470 (2)	0.0085 (5)	

### Atomic displacement parameters (Å^2^)

**Table:**

	*U*^11^	*U*^22^	*U*^33^	*U*^12^	*U*^13^	*U*^23^
Eu	0.00621 (11)	0.00206 (10)	0.00886 (11)	−0.00049 (5)	0.00168 (7)	−0.00068 (5)
Cs	0.02071 (15)	0.01835 (13)	0.01329 (14)	0.00425 (9)	0.00334 (10)	−0.00079 (8)
P1	0.0062 (4)	0.0021 (4)	0.0096 (4)	0.0002 (3)	0.0017 (3)	−0.0003 (3)
P2	0.0059 (4)	0.0031 (4)	0.0094 (4)	−0.0003 (3)	0.0026 (3)	0.0003 (3)
P3	0.0070 (4)	0.0036 (4)	0.0082 (4)	0.0000 (3)	0.0009 (3)	0.0006 (3)
P4	0.0074 (4)	0.0025 (4)	0.0094 (4)	0.0003 (3)	0.0011 (3)	0.0006 (3)
O1	0.0109 (12)	0.0084 (11)	0.0128 (12)	−0.0017 (9)	0.0056 (10)	0.0008 (9)
O2	0.0074 (11)	0.0076 (11)	0.0101 (12)	0.0008 (9)	0.0025 (10)	0.0017 (9)
O3	0.0114 (12)	0.0069 (11)	0.0104 (12)	−0.0026 (9)	0.0020 (10)	−0.0032 (9)
O4	0.0085 (12)	0.0052 (11)	0.0203 (14)	−0.0010 (9)	0.0048 (10)	−0.0013 (10)
O5	0.0105 (13)	0.0127 (12)	0.0133 (13)	−0.0055 (10)	0.0032 (10)	−0.0025 (10)
O6	0.0111 (13)	0.0081 (12)	0.0127 (13)	−0.0027 (9)	0.0016 (10)	−0.0037 (9)
O7	0.0109 (13)	0.0108 (11)	0.0116 (13)	−0.0016 (9)	0.0035 (10)	−0.0014 (9)
O8	0.0092 (12)	0.0066 (11)	0.0130 (13)	0.0032 (9)	0.0028 (10)	0.0032 (9)
O9	0.0189 (13)	0.0047 (11)	0.0104 (12)	−0.0017 (10)	0.0027 (10)	−0.0002 (9)
O10	0.0085 (12)	0.0084 (12)	0.0177 (13)	0.0018 (9)	0.0015 (10)	−0.0041 (10)
O11	0.0165 (13)	0.0037 (11)	0.0152 (13)	−0.0027 (9)	0.0063 (10)	−0.0019 (9)
O12	0.0053 (11)	0.0028 (10)	0.0171 (13)	−0.0006 (8)	0.0024 (10)	−0.0022 (9)

### Geometric parameters (Å, °)

**Table:**

Eu—O5	2.344 (3)	P2—O1	1.485 (2)
Eu—O11	2.360 (2)	P2—O3	1.495 (3)
Eu—O4	2.379 (2)	P2—O2	1.605 (3)
Eu—O7	2.408 (3)	P2—O12	1.609 (2)
Eu—O10	2.413 (2)	P2—Eu^ii^	3.5752 (9)
Eu—O1^i^	2.416 (2)	P3—O5^viii^	1.479 (3)
Eu—O3^ii^	2.445 (2)	P3—O6	1.492 (3)
Eu—O6	2.471 (3)	P3—O2^ix^	1.607 (2)
Eu—P2^ii^	3.5752 (9)	P3—O9^x^	1.609 (3)
Eu—P3	3.5994 (9)	P4—O10^iv^	1.481 (3)
Eu—Cs	4.1024 (3)	P4—O11^xi^	1.484 (3)
Eu—Cs^iii^	4.4773 (4)	P4—O9	1.594 (3)
Cs—O3	3.083 (2)	P4—O8	1.605 (3)
Cs—O11^iv^	3.089 (2)	P4—Cs^iv^	3.7102 (9)
Cs—O7^iv^	3.093 (3)	O1—Eu^viii^	2.416 (2)
Cs—O1	3.114 (3)	O2—P3^v^	1.607 (2)
Cs—O4	3.224 (3)	O3—Eu^ii^	2.445 (2)
Cs—O8	3.249 (3)	O3—Cs^ii^	3.693 (3)
Cs—O12^v^	3.315 (2)	O5—P3^i^	1.479 (3)
Cs—O10	3.354 (3)	O7—P1^vi^	1.479 (3)
Cs—O6	3.399 (3)	O7—Cs^iii^	3.093 (3)
Cs—O9	3.517 (2)	O9—P3^xi^	1.609 (3)
Cs—O10^iv^	3.586 (3)	O10—P4^iii^	1.481 (3)
Cs—P2	3.6075 (9)	O10—Cs^iii^	3.586 (3)
P1—O7^vi^	1.479 (3)	O11—P4^x^	1.484 (3)
P1—O4	1.485 (2)	O11—Cs^iii^	3.089 (2)
P1—O8	1.611 (3)	O12—P1^ii^	1.612 (2)
P1—O12^ii^	1.612 (2)	O12—Cs^ix^	3.315 (2)
P1—Cs^vii^	3.7970 (9)		
			
O5—Eu—O11	118.20 (9)	O4—Cs—O10^iv^	60.36 (6)
O5—Eu—O4	79.62 (9)	O8—Cs—O10^iv^	42.64 (6)
O11—Eu—O4	141.79 (8)	O12^v^—Cs—O10^iv^	79.81 (6)
O5—Eu—O7	70.90 (9)	O10—Cs—O10^iv^	80.580 (8)
O11—Eu—O7	71.60 (8)	O6—Cs—O10^iv^	128.21 (6)
O4—Eu—O7	85.04 (9)	O9—Cs—O10^iv^	41.28 (6)
O5—Eu—O10	139.03 (9)	O3—Cs—P2	24.24 (5)
O11—Eu—O10	75.15 (9)	O11^iv^—Cs—P2	120.18 (5)
O4—Eu—O10	70.81 (8)	O7^iv^—Cs—P2	90.79 (5)
O7—Eu—O10	78.72 (9)	O1—Cs—P2	24.12 (4)
O5—Eu—O1^i^	78.33 (9)	O4—Cs—P2	156.80 (5)
O11—Eu—O1^i^	75.98 (8)	O8—Cs—P2	145.49 (5)
O4—Eu—O1^i^	142.22 (8)	O12^v^—Cs—P2	65.31 (5)
O7—Eu—O1^i^	115.59 (9)	O10—Cs—P2	121.11 (5)
O10—Eu—O1^i^	141.10 (9)	O6—Cs—P2	85.97 (5)
O5—Eu—O3^ii^	75.28 (8)	O9—Cs—P2	113.94 (4)
O11—Eu—O3^ii^	144.64 (8)	O10^iv^—Cs—P2	142.83 (4)
O4—Eu—O3^ii^	69.56 (8)	O7^vi^—P1—O4	121.10 (15)
O7—Eu—O3^ii^	140.72 (8)	O7^vi^—P1—O8	110.06 (14)
O10—Eu—O3^ii^	117.57 (9)	O4—P1—O8	108.01 (15)
O1^i^—Eu—O3^ii^	75.37 (8)	O7^vi^—P1—O12^ii^	106.09 (14)
O5—Eu—O6	142.87 (8)	O4—P1—O12^ii^	110.89 (13)
O11—Eu—O6	79.39 (9)	O8—P1—O12^ii^	98.32 (13)
O4—Eu—O6	107.21 (9)	O7^vi^—P1—Cs^vii^	51.19 (10)
O7—Eu—O6	144.72 (9)	O4—P1—Cs^vii^	157.90 (11)
O10—Eu—O6	74.71 (9)	O8—P1—Cs^vii^	93.73 (10)
O1^i^—Eu—O6	74.81 (9)	O12^ii^—P1—Cs^vii^	60.52 (9)
O3^ii^—Eu—O6	73.47 (8)	O7^vi^—P1—Cs	152.14 (11)
O5—Eu—P2^ii^	57.86 (6)	O4—P1—Cs	54.51 (10)
O11—Eu—P2^ii^	156.53 (6)	O8—P1—Cs	56.39 (10)
O4—Eu—P2^ii^	61.64 (6)	O12^ii^—P1—Cs	100.29 (10)
O7—Eu—P2^ii^	121.82 (6)	Cs^vii^—P1—Cs	143.55 (2)
O10—Eu—P2^ii^	124.21 (6)	O1—P2—O3	116.76 (15)
O1^i^—Eu—P2^ii^	80.66 (6)	O1—P2—O2	107.71 (14)
O3^ii^—Eu—P2^ii^	19.06 (6)	O3—P2—O2	111.15 (14)
O6—Eu—P2^ii^	92.51 (6)	O1—P2—O12	108.98 (14)
O5—Eu—P3	156.63 (6)	O3—P2—O12	108.82 (14)
O11—Eu—P3	62.28 (6)	O2—P2—O12	102.45 (13)
O4—Eu—P3	114.99 (6)	O1—P2—Eu^ii^	149.02 (11)
O7—Eu—P3	126.11 (6)	O3—P2—Eu^ii^	32.27 (10)
O10—Eu—P3	64.27 (6)	O2—P2—Eu^ii^	90.91 (9)
O1^i^—Eu—P3	79.40 (6)	O12—P2—Eu^ii^	90.11 (9)
O3^ii^—Eu—P3	92.32 (6)	O1—P2—Cs	58.96 (10)
O6—Eu—P3	18.85 (6)	O3—P2—Cs	57.81 (10)
P2^ii^—Eu—P3	111.36 (2)	O2—P2—Cs	130.28 (9)
O5—Eu—Cs	123.34 (6)	O12—P2—Cs	127.26 (10)
O11—Eu—Cs	118.09 (6)	Eu^ii^—P2—Cs	90.08 (2)
O4—Eu—Cs	51.71 (6)	O5^viii^—P3—O6	118.20 (15)
O7—Eu—Cs	122.82 (6)	O5^viii^—P3—O2^ix^	107.64 (14)
O10—Eu—Cs	54.82 (6)	O6—P3—O2^ix^	110.36 (14)
O1^i^—Eu—Cs	121.45 (6)	O5^viii^—P3—O9^x^	109.94 (14)
O3^ii^—Eu—Cs	62.80 (6)	O6—P3—O9^x^	110.66 (15)
O6—Eu—Cs	55.84 (6)	O2^ix^—P3—O9^x^	98.14 (13)
P2^ii^—Eu—Cs	72.864 (15)	O5^viii^—P3—Eu	109.19 (11)
P3—Eu—Cs	64.255 (15)	O6—P3—Eu	32.35 (10)
O5—Eu—Cs^iii^	110.19 (6)	O2^ix^—P3—Eu	138.19 (9)
O11—Eu—Cs^iii^	40.47 (6)	O9^x^—P3—Eu	87.37 (10)
O4—Eu—Cs^iii^	103.03 (6)	O5^viii^—P3—Cs	69.17 (11)
O7—Eu—Cs^iii^	40.98 (6)	O6—P3—Cs	51.61 (10)
O10—Eu—Cs^iii^	52.97 (6)	O2^ix^—P3—Cs	113.51 (9)
O1^i^—Eu—Cs^iii^	113.24 (6)	O9^x^—P3—Cs	147.29 (10)
O3^ii^—Eu—Cs^iii^	170.28 (6)	Eu—P3—Cs	63.810 (14)
O6—Eu—Cs^iii^	103.77 (6)	O10^iv^—P4—O11^xi^	118.71 (15)
P2^ii^—Eu—Cs^iii^	160.739 (15)	O10^iv^—P4—O9	108.98 (15)
P3—Eu—Cs^iii^	85.139 (15)	O11^xi^—P4—O9	110.52 (14)
Cs—Eu—Cs^iii^	107.796 (7)	O10^iv^—P4—O8	108.41 (14)
O3—Cs—O11^iv^	140.63 (7)	O11^xi^—P4—O8	109.63 (15)
O3—Cs—O7^iv^	96.87 (7)	O9—P4—O8	98.71 (14)
O11^iv^—Cs—O7^iv^	53.65 (6)	O10^iv^—P4—Cs^iv^	64.61 (11)
O3—Cs—O1	48.36 (6)	O11^xi^—P4—Cs^iv^	54.28 (10)
O11^iv^—Cs—O1	98.19 (7)	O9—P4—Cs^iv^	127.28 (10)
O7^iv^—Cs—O1	84.17 (7)	O8—P4—Cs^iv^	133.76 (10)
O3—Cs—O4	147.89 (6)	O10^iv^—P4—Cs	71.77 (11)
O11^iv^—Cs—O4	71.25 (6)	O11^xi^—P4—Cs	167.85 (11)
O7^iv^—Cs—O4	111.18 (6)	O9—P4—Cs	68.87 (9)
O1—Cs—O4	146.42 (6)	O8—P4—Cs	59.27 (10)
O3—Cs—O8	121.57 (6)	Cs^iv^—P4—Cs	136.34 (2)
O11^iv^—Cs—O8	88.01 (6)	P2—O1—Eu^viii^	149.05 (16)
O7^iv^—Cs—O8	91.30 (6)	P2—O1—Cs	96.92 (12)
O1—Cs—O8	167.93 (6)	Eu^viii^—O1—Cs	113.90 (9)
O4—Cs—O8	45.55 (6)	P2—O2—P3^v^	125.03 (15)
O3—Cs—O12^v^	58.05 (6)	P2—O3—Eu^ii^	128.67 (14)
O11^iv^—Cs—O12^v^	98.84 (6)	P2—O3—Cs	97.95 (11)
O7^iv^—Cs—O12^v^	45.19 (6)	Eu^ii^—O3—Cs	133.38 (9)
O1—Cs—O12^v^	76.10 (6)	P2—O3—Cs^ii^	117.46 (12)
O4—Cs—O12^v^	136.03 (6)	Eu^ii^—O3—Cs^ii^	81.12 (7)
O8—Cs—O12^v^	92.77 (6)	Cs—O3—Cs^ii^	76.82 (6)
O3—Cs—O10	141.66 (6)	P1—O4—Eu	139.70 (15)
O11^iv^—Cs—O10	46.43 (6)	P1—O4—Cs	103.46 (12)
O7^iv^—Cs—O10	99.72 (6)	Eu—O4—Cs	92.90 (8)
O1—Cs—O10	99.37 (6)	P3^i^—O5—Eu	146.62 (16)
O4—Cs—O10	49.90 (6)	P3—O6—Eu	128.80 (15)
O8—Cs—O10	92.42 (6)	P3—O6—Cs	108.25 (13)
O12^v^—Cs—O10	144.62 (6)	Eu—O6—Cs	87.17 (7)
O3—Cs—O6	95.38 (6)	P1^vi^—O7—Eu	142.64 (15)
O11^iv^—Cs—O6	96.23 (6)	P1^vi^—O7—Cs^iii^	106.94 (12)
O7^iv^—Cs—O6	142.10 (6)	Eu—O7—Cs^iii^	108.31 (9)
O1—Cs—O6	77.64 (6)	P4—O8—P1	129.62 (16)
O4—Cs—O6	72.20 (6)	P4—O8—Cs	95.59 (11)
O8—Cs—O6	112.13 (6)	P1—O8—Cs	99.22 (11)
O12^v^—Cs—O6	151.27 (6)	P4—O9—P3^xi^	134.46 (17)
O10—Cs—O6	52.07 (6)	P4—O9—Cs	86.11 (10)
O3—Cs—O9	97.16 (6)	P3^xi^—O9—Cs	139.40 (13)
O11^iv^—Cs—O9	88.72 (6)	P4^iii^—O10—Eu	149.26 (16)
O7^iv^—Cs—O9	58.85 (6)	P4^iii^—O10—Cs	91.87 (12)
O1—Cs—O9	127.52 (6)	Eu—O10—Cs	89.14 (7)
O4—Cs—O9	84.95 (6)	P4^iii^—O10—Cs^iii^	85.12 (11)
O8—Cs—O9	41.82 (6)	Eu—O10—Cs^iii^	94.52 (8)
O12^v^—Cs—O9	51.48 (6)	Cs—O10—Cs^iii^	176.32 (8)
O10—Cs—O9	121.00 (6)	P4^x^—O11—Eu	139.40 (15)
O6—Cs—O9	153.49 (6)	P4^x^—O11—Cs^iii^	102.77 (12)
O3—Cs—O10^iv^	136.01 (6)	Eu—O11—Cs^iii^	109.80 (8)
O11^iv^—Cs—O10^iv^	51.06 (6)	P2—O12—P1^ii^	131.54 (16)
O7^iv^—Cs—O10^iv^	53.89 (6)	P2—O12—Cs^ix^	132.22 (12)
O1—Cs—O10^iv^	136.86 (6)	P1^ii^—O12—Cs^ix^	94.44 (10)

Symmetry codes: (i) *x*−1/2, −*y*+1/2, *z*−1/2; (ii) −*x*+1, −*y*, −*z*+1; (iii) −*x*+3/2, *y*+1/2, −*z*+1/2; (iv) −*x*+3/2, *y*−1/2, −*z*+1/2; (v) −*x*+3/2, *y*−1/2, −*z*+3/2; (vi) −*x*+1, −*y*, −*z*; (vii) *x*−1/2, −*y*−1/2, *z*−1/2; (viii) *x*+1/2, −*y*+1/2, *z*+1/2; (ix) −*x*+3/2, *y*+1/2, −*z*+3/2; (x) *x*, *y*+1, *z*; (xi) *x*, *y*−1, *z*.
